# Class A Penicillin-Binding Protein-Mediated Cell Wall Synthesis Promotes Structural Integrity during Peptidoglycan Endopeptidase Insufficiency in Vibrio cholerae

**DOI:** 10.1128/mBio.03596-20

**Published:** 2021-04-06

**Authors:** Shannon G. Murphy, Andrew N. Murtha, Ziyi Zhao, Laura Alvarez, Peter Diebold, Jung-Ho Shin, Michael S. VanNieuwenhze, Felipe Cava, Tobias Dörr

**Affiliations:** a Weill Institute for Cell and Molecular Biology, Cornell University, Ithaca, New York, USA; b Department of Microbiology, Cornell University, Ithaca, New York, USA; c The Laboratory for Molecular Infection Medicine Sweden (MIMS), Department of Molecular Biology, Umeå University, Umeå, Sweden; d Department of Molecular and Cellular Biochemistry and Department of Biology, Indiana University, Bloomington, Indiana, USA; e Cornell Institute of Host-Microbe Interactions and Disease, Cornell University, Ithaca, New York, USA; Indiana University Bloomington; Fred Hutchinson Cancer Research Center

**Keywords:** peptidoglycan, autolysin, endopeptidase, M23, LysM, penicillin-binding protein, mreB, cell wall, penicillin-binding proteins

## Abstract

The bacterial cell wall is composed primarily of peptidoglycan (PG), a poly-aminosugar that is essential to sustain cell shape, growth, and structural integrity. PG is synthesized by class A/B penicillin-binding proteins (a/bPBPs) and shape, elongation, division, and sporulation (SEDS) proteins like RodA (as part of the Rod system cell elongation machinery) and degraded by “autolytic” enzymes to accommodate growth processes. It is thought that autolysins (particularly endopeptidases [EPs]) are required for PG synthesis and incorporation by creating gaps that are patched and paved by PG synthases, but the exact relationship between autolysins and PG synthesis remains incompletely understood. Here, we have probed the consequences of EP depletion for PG synthesis in the diarrheal pathogen Vibrio cholerae. We found that EP depletion resulted in severe morphological and division defects, but these cells continued to increase in mass and aberrantly incorporated new cell wall material. Mass increase proceeded in the presence of Rod system inhibitors, but cells lysed upon inhibition of aPBPs, suggesting that aPBPs are required for structural integrity under these conditions. The Rod system, although not essential for the observed mass increase, remained functional even after prolonged EP depletion. Last, heterologous expression of an EP from Neisseria gonorrhoeae fully complemented growth and morphology of an EP-insufficient V. cholerae, highlighting the possibility that the PG synthases may not necessarily function via direct interaction with EPs. Overall, our findings suggest that during EP insufficiency in V. cholerae, aPBPs become essential for structural integrity while the Rod system is unable to promote proper cell expansion.

## Abstract (precis)

Synthesis and turnover of the bacterial cell wall must be tightly coordinated to avoid structural integrity failure and cell death. Details of this coordination are poorly understood, particularly if and how cell wall turnover enzymes are required for the activity of the different cell wall synthesis machines, the aPBPs and the Rod system.

## INTRODUCTION

Most bacteria elaborate a cell wall composed of peptidoglycan (PG), which consists of polymerized *N*-acetylglucosamine-*N*-acetyl muramic acid (poly-GlcNAc-MurNAc) dimers. These polymerized strands are covalently linked to each other via their oligopeptide side stems extending from the MurNAc residues; the degree of cross-linking varies with bacterial species and growth conditions (1–3). As such, PG encases the cell in a net-like structure that functions to maintain the high intracellular pressure accumulating in most bacteria and thus to prevent the cell from lysing. In concert with maintenance of structural integrity, PG has to accommodate growth processes (cell elongation and size expansion) and is therefore constantly degraded and resynthesized (4–6).

In many rod-shaped Gram-negative bacteria, cell wall synthesis during cell elongation is mediated by two types of cell wall synthase complexes: the Rod complex (which includes the glycosyltransferase RodA in conjunction with a class B penicillin-binding protein [bPBP] and accessory proteins) and the class A PBPs (aPBPs) in conjunction with their lipoprotein activators (7–12). The differential physiological roles of these PG synthases have only recently begun to be dissected (13–15) but remain incompletely understood. Vibrio cholerae encodes a “canonical” Rod system consisting of RodA, bPBP2, MreBCD, and RodZ, each of which is essential for viability (16). The two aPBPs are, as in other Gram-negative bacteria, conditionally essential (i.e., at least one has to be present for viability) with overlapping, but also partially distinct functions: aPBP1a is the major class A PBP (12), with a more pronounced role in cell elongation, while aPBP1b appears to be more important for cell division (11).

Cell wall degradation, on the other hand, is mediated by a plethora of so-called “autolysins,” i.e., enzymes with the capability to break bonds in the PG sacculus (17–19). Members of one such group of autolysins, the endopeptidases (EPs), cleave the oligopeptide cross-links between PG strands, presumably to allow for insertion of new PG material during cell elongation (20–23). To ensure structural integrity, EP-mediated cell wall cleavage and Rod- and/or aPBP-mediated resynthesis should logically be tightly coordinated, and this has indeed been demonstrated for cell elongation in Gram-positive bacteria (24–27). When the putative coordination is perturbed (e.g., after exposure to a cell wall synthesis inhibitor), PG structural integrity often catastrophically fails, and cells die (28); this is one of the reasons why cell wall synthesis inhibitors (e.g., the β-lactams) rank highly among our most powerful antimicrobials (29). EPs in particular are a double-edged sword as they can both promote cell wall synthesis (30) and play major roles in cell wall cleavage after β-lactam exposure (31, 32). However, how EPs are regulated has only begun to be unravelled (33–36), and at least in Gram-negative bacteria, we lack a complete understanding of how EP cleavage activity relates to PG synthesis by the two distinct cell wall synthase complexes.

Several models have been advanced to explain coordination of synthesis and degradation, with a prominent model being a “make-before-break” mechanism, where a nascent PG layer scaffold is elaborated parallel to an existing one, followed by cleavage of the old material that has been relieved of its critical structural function through this nascent PG load-bearing stabilizer (37, 38). Alternatively, PG might be able to sustain several cleavage events without experiencing catastrophic structural failure, obviating the need for any coordination between synthesis and degradation for as long as the Rod system and/or aPBPs are efficient enough in recognizing gaps in PG, for example, through interaction with their cognate outer membrane (OM)-localized activators in the case of the aPBPs (a “break-before-make” model).

Here, we show that in the cholera pathogen Vibrio cholerae, EP activity is not required for cell wall synthesis *per se*. During EP insufficiency, mass and PG accumulation continue in the presence of Rod system inhibitors but stop upon inhibition of aPBPs. However, the Rod system continues directed motion for extended periods of EP depletion. Last, a heterologously expressed EP can fully complement growth and morphology of an EP-deficient V. cholerae strain. Our data suggest that aPBP activity does not require wild-type levels of cross-link cleavage for PG incorporation (and consequently cell expansion), while the Rod system relies more on EP activity to contribute substantially to proper rod-shaped growth and cell expansion. Our cross-species complementation experiments intriguingly raise the possibility that direct coordination between EPs and cell wall synthases might not be necessary at all, at least under standard laboratory growth conditions.

## RESULTS

### V. cholerae continues to increase in mass during endopeptidase insufficiency.

**FIG 1:**
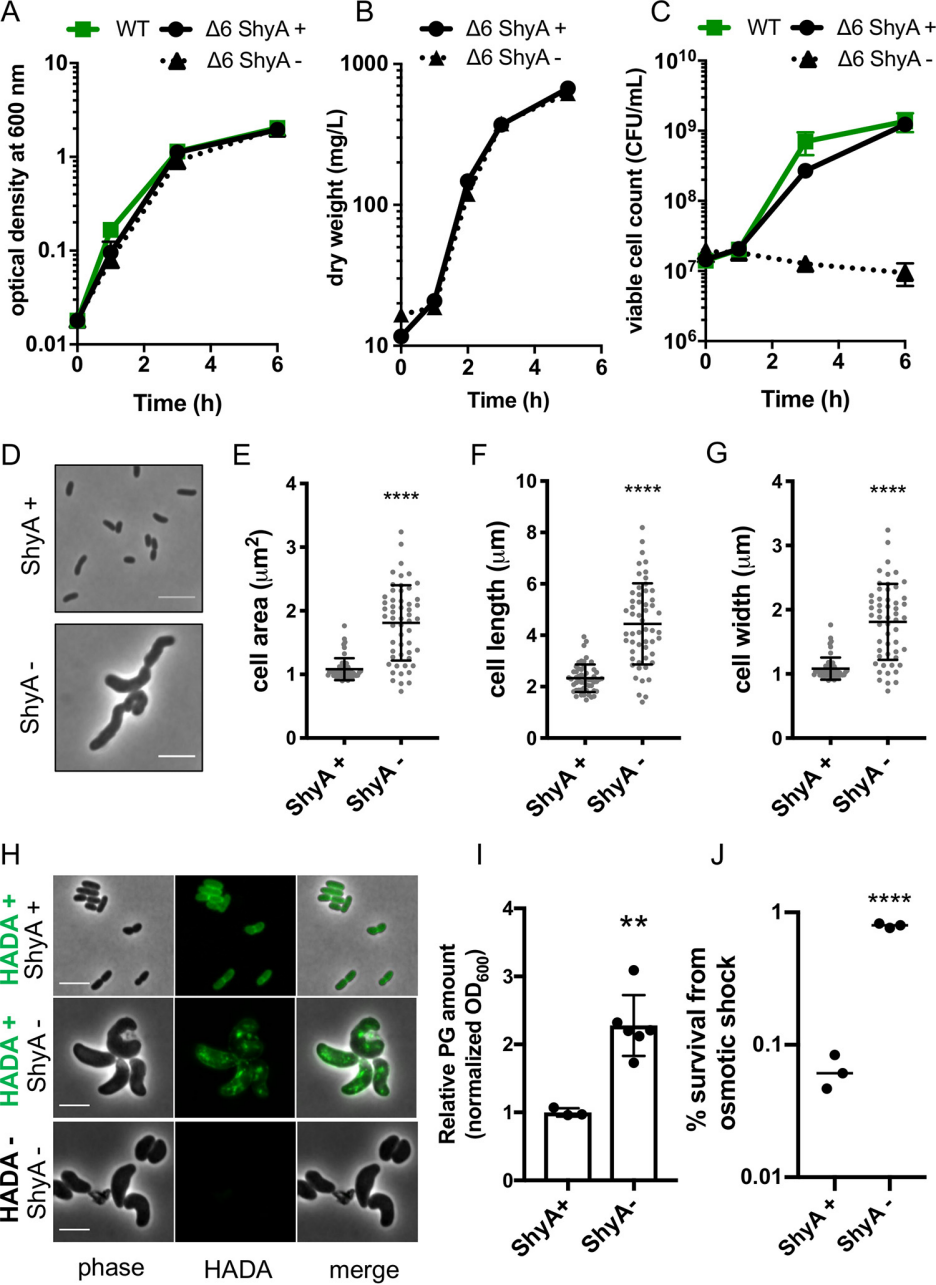
Cell mass increase and cell wall incorporation continue during EP insufficiency. (A to J) Overnight cultures of the Δ6 endo (Δ*shyABC Δvc1537 ΔtagE1*,*2* P_IPTG_::*shyA*) strain grown in the presence of IPTG (200 μM) were washed twice and diluted 100-fold into growth medium with IPTG (ShyA +) or without IPTG (ShyA -). At the indicated time points, optical density (OD_600_) (A), dry mass (mg/liter) (B), and viable cell counts (CFU/ml) (C) were measured. Data are means of at least three biological replicates, and error bars represent standard deviations. (D to G) Cells were imaged at 3 h, and representative cells are shown in panel D. ImageJ was used to measure cell area (E), cell length (F), and cell width (G). Raw data points are shown, and error bars represent standard deviations. Asterisks denote statistical difference relative to ShyA + via a Mann-Whitney test (****, *P* < 0.0001). (H) Δ6 endo strain was grown in the presence of HADA (100 μM) for 3 h, washed twice, and then imaged. All bars, 5 μm. (I) After 2 h of growth, relative PG content of Δ6 endo strain (normalized to OD_600_) was measured via UPLC analysis (see Materials and Methods for details). Bar graphs show data normalized to Shy A+. Error bars represent standard deviations of three biological replicates, and asterisks denote statistical difference via unpaired *t* test (**, *P* < 0.01). (J) After 3 h of growth, cells were pelleted and resuspended in 20 mM NaCl (osmotic shock treatment) for 5 min. Shock treatment was stopped by adding PBS to 180 mM. Percent survival is CFU/ml after treatment divided by CFU/ml before treatment. Raw data points of three biological replicates are shown. Asterisks denote statistical difference via unpaired *t* test (****, *P* < 0.0001).

Endopeptidase depletion was previously shown to preclude insertion of new cell wall material in Escherichia coli, resulting in rapid cell lysis (23). In contrast, we noticed that EP-depleted Vibrio cholerae did not lyse, even in the absence of all six of its major d,d-EPs. This Δ6 endo strain (Δ*shyA ΔshyB ΔshyC Δvc1537 Δvc0843 Δvca1043* P_IPTG_:*shyA*) has the remaining, conditionally essential EP ShyA under the control of an isopropyl-β-d-thiogalactopyranoside (IPTG)-inducible promoter and is thus suitable for depletion experiments. Upon growing the Δ6 endo strain in the absence of inducer for 2 h, ShyA was reduced to less than 10% of initial levels (see Fig. S1A and B in the supplemental material), and the percentage of PG cross-links was significantly increased (35.6% ± 2.1% after depletion compared to 31.6% ± 0.6% when *shyA* is expressed) (Fig. S1C and D). In this state of EP insufficiency, cell mass measured by either optical density (Fig. 1A) or bacterial dry weight (Fig. 1B) continued to increase at a rate similar to when *shyA* was expressed. When we plated these cells on solid medium containing inducer, however, we found that the number of viable cells (CFU per milliliter [CFU/ml]) in the EP-depleted culture remained static or declined slightly (Fig. 1C). This indicates that EP-insufficient cells are not readily dividing but are able to recover and form colonies on a plate if resupplied with inducer (see Movie S1 in the supplemental material). We have previously shown that EP depletion in the Δ6 endo strain results in a dramatic increase in cell size and ultimately the generation of giant, bulky, and contorted cells (36). Here we have quantified these morphology defects and shown that ShyA-depleted Δ6 endo cells have significantly greater cell areas, lengths, and widths (Fig. 1D to G). Overall, these observations show that EP-insufficient V. cholerae cells suffer severe morphology and division defects but still increase in mass and retain structural integrity and viability.

10.1128/mBio.03596-20.2 MOVIE S1 Time-lapse microscopy of Δ6 endo mutant recovering from *shyA* depletion. *shyA* was depleted from the Δ6 endo mutant for 3 h in LB. The ShyA-depleted cells were spotted onto an LB agarose pad (0.8% [wt/vol] agarose) containing 500 μM IPTG to induce *shyA* expression. Cells were incubated at 37°C, and images were taken at 10-min intervals for 8 h. Time-lapse frames were stabilized using ImageJ plugin “StackReg.” Movie S1, AVI file, 0.8 MB Copyright © 2021 Murphy et al. 2021 Murphy et al. https://creativecommons.org/licenses/by/4.0/ This content is distributed under the terms of the Creative Commons Attribution 4.0 International license.

10.1128/mBio.03596-20.5 FIG S1 ShyA levels after depletion in a Δ6 endopeptidase mutant and its effect on growth, survival, and PG composition. (A) Wild-type, Δ6 endo, and Δ*shyA* strains were grown overnight in LB broth containing inducer (IPTG, 200 μM) and then washed three times with fresh LB. Cells were then diluted 100-fold into 150 ml prewarmed LB medium without inducer for ShyA depletion. Samples were collected at indicated time points. For Western blotting, cell extracts (adjusted to same protein concentration) were separated on 10% SDS-PAGE gels and subjected to Western blot analysis using ShyA polyclonal antibody. (B) ShyA band intensities were quantified (ImageJ), and the intensity value of the nonspecific background band detected in the Δ*shyA* mutant was subtracted. Residual ShyA protein levels were normalized to nondepleted ShyA at 0 h (100%). Data are averages of three biological replicates, and error bars represent standard deviations. (C and D) Δ6 endo cells grown for 2 h in the presence (ShyA +) or absence (ShyA -) of inducer were harvested for PG content analysis (see Materials and Methods). (C) PG chromatograms and (D) a table showing the relative molar abundance (as a percentage) of monomers, dimers, and trimers are shown. Standard deviations are shown. Crosslink percentage is the proportion of crosslinked peptide side chains (calculated based on dimer and trimer content) and anhydro muropeptides (with a residue of (1-6 anhydro) N-acetyl muramic acid, abbreviated “Anh”) are the terminal subunits of the sugar chains and hence used to calculate the chain length. Values are means of three biological replicates. Percent change was calculated relative to the IPTG-treated sample, and *P* values were generated using a multiple-comparison *t* test (****, *P* < 0.0001; ***, *P* < 0.001, ** *P* < 0.01, * < 0.05). (E and F) WT, N16961 Δ6 endo, and E7946 Δ9 endo (*mreB*::*mreB*msfGFP^sw^) were treated as described in the legend to Fig. 1. At the indicated time points, OD_600_ (E) was measured via spectrophotometry, and cells were diluted serially onto LB IPTG (200 μM) plates to determine colony-forming units (CFU) per milliliter (F). Note that data for WT and Δ6 endo strains are reproduced from Fig. 1A and B. Data are averages of three biological replicates; error bars represent standard deviations. Download FIG S1, TIF file, 0.4 MB. Copyright © 2021 Murphy et al. 2021 Murphy et al. https://creativecommons.org/licenses/by/4.0/ This content is distributed under the terms of the Creative Commons Attribution 4.0 International license.

To rule out contributions from other predicted endopeptidases, we additionally deleted the genes encoding PBP4, PBP7, and VC1269 (which have predicted EP activity but are, based on work in E. coli, not required for growth and cell elongation [39, 40]). These gene deletions did not appreciably affect mass increase (except for a slight decrease in final yield after 6 h; for an unknown reason, the strain also exhibited a more pronounced drop in CFU/ml than Δ6 endo) (Fig. S1E and F), demonstrating that the mass increase phenotype did not simply reflect the ability of these putative EPs to substitute for ShyA.

### Cell wall incorporation continues during EP insufficiency.

**FIG 2:**
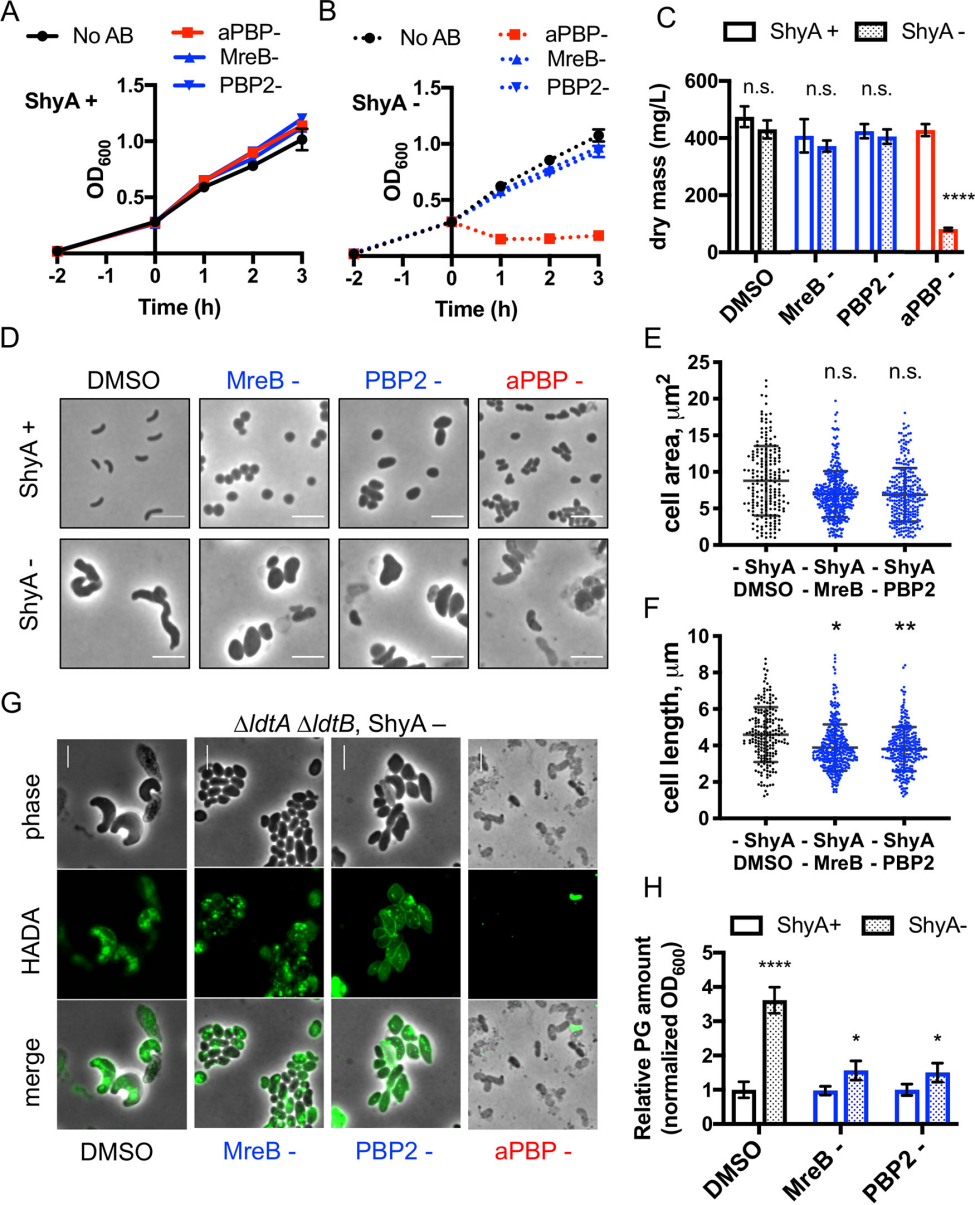
Cell mass increase and PG incorporation during EP insufficiency relies on aPBP activity. Δ6 endo grown overnight in IPTG (200 μM) was washed twice and diluted 100-fold into fresh medium with inducer (ShyA+) (A) or without inducer (B) (ShyA -) (B). After 2 h of growth, either vehicle (0.1% DMSO, shown in black), the aPBP inhibitor moenomycin (aPBP-, 10 μg ml^−1^, 8× MIC, shown in red), the MreB inhibitor MP265 (MreB-, 300 μM, 15× MIC, shown in blue), or the PBP2 inhibitor amdinocillin (PBP2-, 10 μg ml^−1^, 20× MIC, shown in blue) were added. At the indicated time points, OD_600_ was measured; data are averages of three biological replicates, and error bars represent standard deviations. (C) At 2 h after drug treatment, 120 ml of cells was harvested and dried on a heat block (65°C) until the mass steadied (∼24 h). Dry mass values were transformed into milligrams per liter. Data are averages of three biological replicates, and error bars represent standard deviations. Asterisks denote statistical difference via paired multiple *t* tests (****, *P* < 0.0001). n.s., not significant. (D) Representative cell morphologies 2 h after drug addition are shown. (E) Cell area (square micrometers) and (F) cell length (micrometer) were measured in ImageJ. Raw data points are shown. Asterisks denote statistical difference via Kruskal-Wallis test (**, *P* < 0.01; *, *P* < 0.05). (G) An overnight culture of the Δ6 endo Δ*ldtA ΔldtB* strain was diluted into medium without inducer. After 2 h of ShyA depletion, HADA (100 μM) was added for another 1 h. Cells were then washed twice and imaged. Antibiotics moenomycin (aPBP -), MP265 (MreB -), and amdinocillin (PBP2 -) were added for 1 h after the 2-h initial depletion, followed by 1-h addition of HADA. All bars, 5 μm. (H) At 2 h after drug treatment, relative PG content of Δ6 endo strain relative to OD_600_ was measured via UPLC analysis (see Materials and Methods for details). Data are normalized to the ShyA+ DMSO sample. Error bars represent standard deviations of three biological replicates. Asterisks denote statistical difference via unpaired *t* tests (****, *P* < 0.0001; *, *P* < 0.05).

We next probed the impact of endopeptidase insufficiency on PG incorporation and composition. To determine whether these enlarged cells elaborated a wild-type PG cell wall, we cultured Δ6 endo in the presence of a fluorescent d-amino acid derivative, HADA (3-[[(7-hydroxy-2-oxo-2*H*-1-benzopyran-3-yl)carbonyl]amino]-d-alanine hydrochloride), as a cell wall stain (41). Addition of HADA to ShyA-replete Δ6 endo cells resulted in an even distribution of staining along the cell wall (Fig. 1H), as expected from wild-type cell wall synthesis. In contrast, depleting ShyA resulted in a strikingly different pattern, where large patches of HADA-reactive material accumulated throughout the cell. In principle, these patches could be a remnant of incompletely degraded cell wall material synthesized before ShyA was completely depleted, or they could reflect the activity of l,d-transpeptidases (which are able to incorporate HADA into the cell wall independent of cell wall synthesis [41, 42]). We repeated the staining experiment in a Δ6 endo strain lacking l,d-transpeptidases (Δ*ldtA ΔldtB*). Following a 2-h depletion, we added HADA for an additional hour; this still revealed an accumulation of aberrant PG patches (Fig. 2G). While accumulation of HADA is difficult to interpret quantitatively (due to multiple possible upstream events that could result in increased HADA signal [43]), these data qualitatively show that during endopeptidase insufficiency, PG synthesis and incorporation continue in an aberrant, nondirectional way.

Quantification of PG confirmed and expanded these observations—after 2 h of ShyA depletion, cells accumulated approximately two- to threefold-more PG than ShyA-replete cells (when normalized to an optical density at 600 nm [OD_600_]) (Fig. 1I). Since these cells are nondividing, we speculate that PG is accumulating within these individual, drastically enlarged ShyA-depleted cells. Consistent with a higher cell wall content of individual cells, ShyA-depleted Δ6 endo cells were almost 10-fold more resistant to osmotic shock treatment (Fig. 1J). Thus, ShyA-depleted Δ6 endo cells not only incorporate PG but retain higher levels of PG than the wild type (WT), possibly reflecting the lack of EP-initiated turnover processes. Similar observations have been made in autolysin-inactivated Bacillus subtilis, a Gram-positive bacterium (27, 44, 45). While we cannot rule out that residual ShyA remains in the cell following depletion (at levels too low to detect above background of the nonspecific band we observed via Western blotting in the same size range as ShyA [Fig. S1A and B]), we can at a minimum conclude that wild-type levels of V. cholerae’s principal EPs are not necessary to facilitate mass increase and incorporation of PG *per se*. Rather, EPs are essential for maintenance of width homeostasis and cell division, and likely key for the proper, directional integration of PG into the V. cholerae sacculus.

### Cell wall incorporation and mass increase in EP-deficient cells rely primarily on aPBPs.

We next addressed whether EP insufficiency affected the two cell wall synthases, the Rod system and the aPBPs, differentially. To this end, we grew the Δ6 endo strain in the presence or absence of inducer for 2 h and then treated with either moenomycin (an aPBP glycosyltransferase inhibitor [46]) or one of two Rod system inhibitors: MP265 (an inhibitor of MreB [47]) or amdinocillin (an inhibitor of PBP2 [48]).

When ShyA was expressed, mass increase (proxied by OD_600_, which correlates strongly with dry weight [Fig. 1AB]) proceeded at similar rates for all antibiotic treatments and the dimethyl sulfoxide (DMSO) control (Fig. 2A), while CFU/ml plateaued in the presence of antibiotic (Fig. S2A). The continued OD_600_ increase upon antibiotic exposure is consistent with our previous observations that V. cholerae (as well as many clinically significant Gram-negative pathogens) is remarkably tolerant to inhibitors of cell wall synthesis (32, 49). Exposure to such agents causes V. cholerae to form cell wall-deficient spheroplasts (in the presence of aPBP inhibitors) or spheroid cells containing cell wall material (in the presence of MreB and PBP2 inhibitors) (32, 49). Importantly, both sphere cell types continue to increase in mass (32, 49) but fail to divide. Thus, OD_600_ continues to increase while CFU/ml remains unchanged.

10.1128/mBio.03596-20.6 FIG S2 Mass increase during EP insufficiency requires aPBPs, whereas the Rod system influences cell morphology. Δ6 endo strain was grown overnight in IPTG (200 μM), washed twice, and diluted 100-fold into fresh medium containing IPTG (ShyA +) (A) or not containing IPTG (ShyA -) (B). After 2 h of EP depletion (dashed vertical line), either no antibiotic (DMSO, 0.1%, shown in black), the aPBP inhibitor moenomycin (aPBP -, 10 μg ml^−1^, 8× MIC, shown in red), the MreB inhibitor MP265 (MreB -, 300 μM, 15× MIC, shown in blue), or the PBP2 inhibitor mecillinam (PBP2-, 10 μg ml^−1^, 20× MIC, shown in blue) were added. At the indicated time points, cells were diluted serially onto LB IPTG (200 μM) plates to determine viable colony-forming units (CFU) per ml (B). Data are averages of three biological replicates; error bars represent standard deviations. (C to E) At 2 h post-drug treatment (+ 2 h), cells were harvested and spotted on a 0.8% agarose pad containing PBS for phase contrast microscopy. ImageJ was used to measure cell area (C), length (D), and width (E) of individual cells (*n* > 200). *P* values were generated using a Kruskal-Wallis multiple-comparison test (****, *P* < 0.0001; ***, *P* < 0.001; ** *P* < 0.01; *, *P* < 0.05). Note that ShyA - data are the same as shown in Fig. 2. (F to H) Cells were grown as described above for panels A and B but treated with DMSO (1%) or the aPBP (pbp1b) inhibitor cefsulodin (1 mg ml^−1^, shown in red). Cells were imaged at 2 h post-drug treatment on a 0.8% agarose pad; representative images are shown (E). All scale bars, 5 μM. At the indicated time points, optical density (OD_600_) (G) and viable cell counts (CFU/ml) (H) were measured. Data are means of at least three biological replicates; error bars represent standard deviations. Download FIG S2, TIF file, 0.4 MB. Copyright © 2021 Murphy et al. 2021 Murphy et al. https://creativecommons.org/licenses/by/4.0/ This content is distributed under the terms of the Creative Commons Attribution 4.0 International license.

Upon ShyA depletion, mass increase (OD_600_) in the presence of MP265 or amdinocillin-treated cells continued at a similar rate compared to untreated and ShyA-replete conditions (Fig. 2B). Measurements of bacterial dry weight at 2 h posttreatment corroborated this finding (Fig. 2C). These data suggest that the Rod system is not required for the observed mass increase in EP-insufficient cells. ShyA depletion still generated enlarged cells in the presence of either Rod system inhibitor; however, the cells were noticeably rounder than untreated cells (cells not treated with ShyA [ShyA- cells]) (Fig. 2D). Cell dimension analysis in ImageJ (50) revealed that neither Rod system inhibitor affected the two-dimensional (2D) area of EP-insufficient cells (Fig. 2E); however, both drugs significantly decreased cell length (Fig. 2F; additional cell size analyses are presented in Fig. S2C to E). Altogether, these data suggest that the Rod system is not required for mass increase but still contributes to cell shape during EP insufficiency. We next visualized and quantified cell wall material in the presence of Rod system inhibitors. After 2 h of treatment with either MP265 or amdinocillin, we observed substantial HADA incorporation in ShyA- cells (Fig. 2G), consistent with our interpretation that the Rod system is not absolutely required for PG synthesis during EP insufficiency. PG quantifications revealed that when EP-insufficient cells were treated with either Rod system inhibitor, they still accumulated more PG than similarly treated cells treated in the presence of ShyA (ShyA+ cells), albeit to a lower degree (Fig. 2H). Importantly, neither treatment resulted in PG degradation.

In striking contrast to Rod system inhibition, aPBP inhibition via moenomycin exposure completely abrogated growth (measured by OD_600_ and dry mass) of ShyA-depleted Δ6 endo cells (Fig. 2B and C). This coincided with accumulation of small cells and debris, indicative of lysis (Fig. 2D). In addition, cell viability declined rapidly in early stages (consistent with our previous observations [32]), though ultimately exhibited levels of survival similar to MP265- or amdinocillin-treated, ShyA-depleted Δ6 endo cells (Fig. S2B). We observed comparable results when the aPBP inhibitor cefsulodin was used at high concentrations (Fig. S2F to H). Notably, aPBP-inhibited cells lacked strong HADA incorporation (Fig. 2H), though these data are difficult to interpret due to the loss of cell wall integrity. However, our data clearly suggest that during EP insufficiency, the aPBPs are required to maintain structural integrity and are major contributors to both mass increase and sustained PG incorporation, while the Rod system primarily influences cell morphology.

### MreB movement continues in EP-insufficient cells.

**FIG 3:**
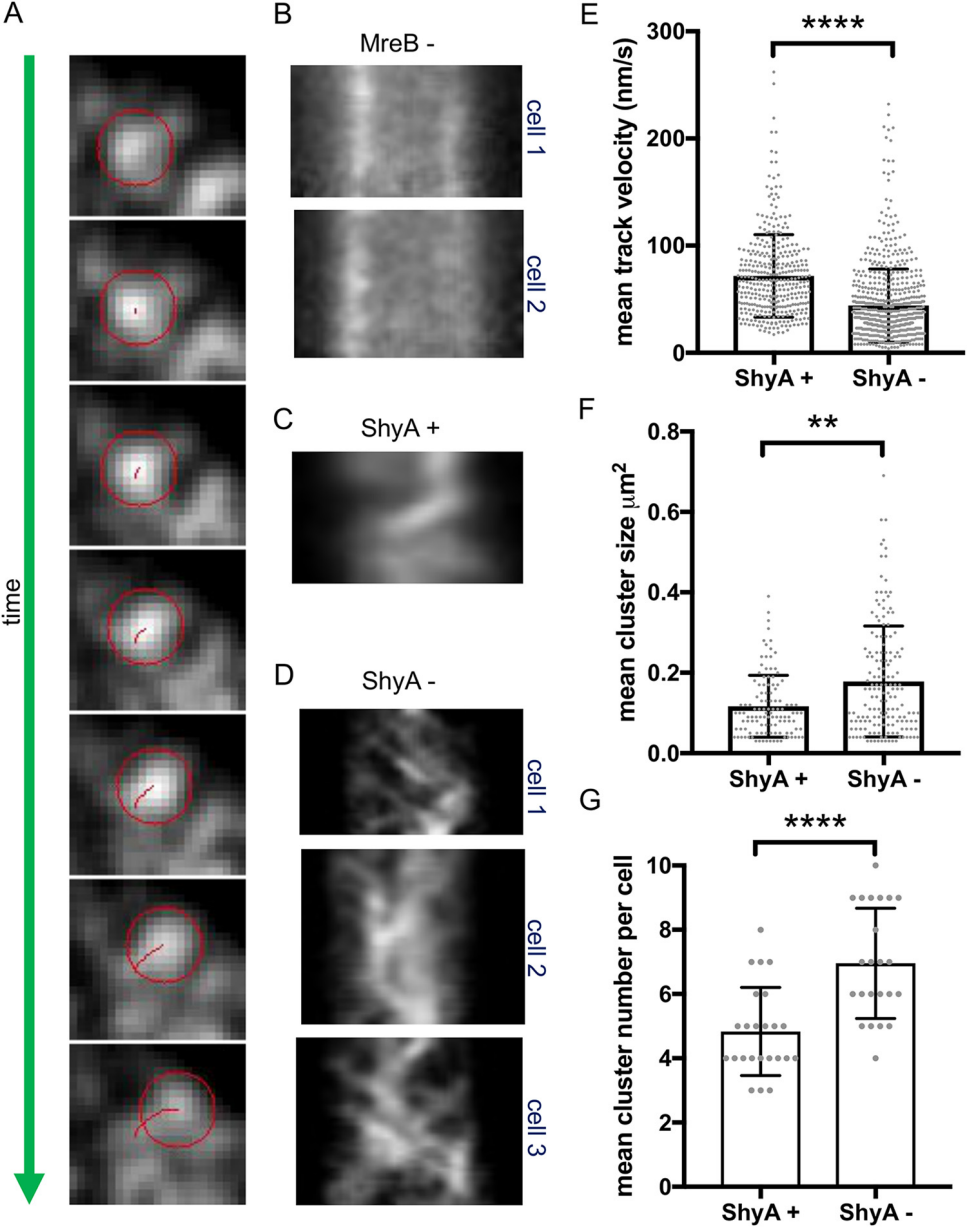
MreB movement continues during EP insufficiency. Δ6 endo (A to D) or Δ8 endo (E to G) strain expressing an mreBmsfGFP^sw^ fusion from its native chromosomal locus was diluted from an overnight culture grown in the presence of IPTG into growth medium without inducer (ShyA -). After 3 h, cells were imaged using epifluorescence microscopy (A to D) or TIRF (E to G). MreB movement was analyzed using Fiji (TrackMate). (A) A representative single moving MreB focus track (red circle) is shown (frames are 2.5 s apart). (B to D) Representative kymographs of MreB foci are shown for cells grown in the presence of the MreB inhibitor MP265 (MreB -) (B), in the presence of inducer (ShyA +) (C), or in the absence of inducer (ShyA -) (D). (E to G) TIRF was used to assess MreB focus velocity (E), mean cluster size (F), and mean cluster number (G). Raw data points are shown, and error bars represent standard deviations. Asterisks denote statistical difference via Mann-Whitney test (**, *P* < 0.01; ****, *P* < 0.0001).

The Rod system, in conjunction with the actin homolog MreB, deposits new cell wall material during cell elongation while performing a rotational movement around the cell, apparently driven by aPBP-independent cell wall synthesis (51–53). Our data indicate that the Rod system plays a minor role during EP insufficiency, so we asked whether EP depletion altered the mobility of Rod complexes similar to what has been observed during inhibition of cell wall synthesis (52). We constructed a functional (Fig. S3A) MreB^msfGFP^ sandwich fusion in a Δ6 endo background and measured MreB^msfGFP^ velocity using epifluorescence and total internal reflection fluorescence (TIRF) microscopy (see Materials and Methods for details). We determined MreB velocities through particle analysis and mean square displacements (MSD) calculations in ImageJ (54, 55). We confirmed that MP265 stopped MreB movement (Fig. 3B) as a positive control, as expected from what has been reported in E. coli. Mean square displacement values indicated mixed populations of diffusive MreB particles and those exhibiting directed motion under both ShyA-replete and -depleted conditions (Fig. S3B and C). Interestingly, MreB movement continued even after 3 h of ShyA depletion (Fig. 3D and Movie S2), albeit with a substantially altered pattern of movement and a reduced velocity (∼44 ± 34 nm/s) compared to ShyA-replete conditions (∼72 ± 38 nm/s) (Fig. 3C). This velocity reduction was statistically significant (*P* < 0.0001, Mann-Whitney test). Our estimates of MreB velocity under ShyA-replete conditions were somewhat higher than what has been reported previously for other bacteria (55 nm/s for B. subtilis [56] and 6.7 ± 2.7 nm/s to 30 nm/s for E. coli [52, 56]), perhaps reflecting species-specific differences or different properties of our sandwich fusion. We additionally determined the MreB cluster size and number using ImageJ TrackMate (54) and particle analysis (57, 58) (see Materials and Methods for details). Interestingly, the average cluster size and number increased under ShyA depletion conditions (Fig. 3F and G and Fig. S3D), suggesting that EP depletion might affect Rod complex assembly dynamics and possibly explain the altered pattern of MreB movement. While we currently do not have a mechanistic explanation for those pattern differences, the key observation here is simply that MreB movement persists upon prolonged EP depletion. We thus conclude that similar to what has been observed in B. subtilis (24), EP insufficiency does not result in immediate inactivation of the Rod system but changes its velocity and potentially the dynamics of its assembly.

10.1128/mBio.03596-20.3 MOVIE S2 mreBmsfGFP movement in Δ6 endopeptidase after 3 h of EP depletion. Cells were treated as described in the legend to Fig. 3. Following depletion, cells were transferred to an agarose pad lacking IPTG and imaged by time-lapse microscopy; images were taken every 2.5 s at 100-ms exposure time. Download Movie S2, AVI file, 0.6 MB. Copyright © 2021 Murphy et al. 2021 Murphy et al. https://creativecommons.org/licenses/by/4.0/ This content is distributed under the terms of the Creative Commons Attribution 4.0 International license.

10.1128/mBio.03596-20.7 FIG S3 Growth rate, mean square displacement analysis, and cluster analysis of the mreBmsfGFP strain. (A) Wild-type E7946 and mreBmsfGFP-containing derivative were grown overnight in LB. Cells were diluted 1,000-fold into fresh medium, and 200 μl of each was loaded into a 100-well plate. Growth of each culture was monitored by optical density at 600 nm (OD_600_) in a Bioscreen C plate reader (Growth Curves America). (B to D) Δ6 endo mreBmsfGFP^sw^ strain was grown with (ShyA +) or without (ShyA -) IPTG and imaged using TIRF microscopy. (B) Example MSD curves for two regions of interest (ROIs) for both the ShyA+ and ShyA- condition. (C) Alpha values and percentages of MreBmsfGFP patches exhibiting directed motion. (D) Representative images of mreBmsfGFP^sw^ clusters in Δ6 endo ShyA+ and ShyA- strains used for analysis. Scale bars, 5 μm. Download FIG S3, TIF file, 0.4 MB. Copyright © 2021 Murphy et al. 2021 Murphy et al. https://creativecommons.org/licenses/by/4.0/ This content is distributed under the terms of the Creative Commons Attribution 4.0 International license.

### Complementation of EP insufficiency in V. cholerae by expression of heterologous EPs.

**FIG 4:**
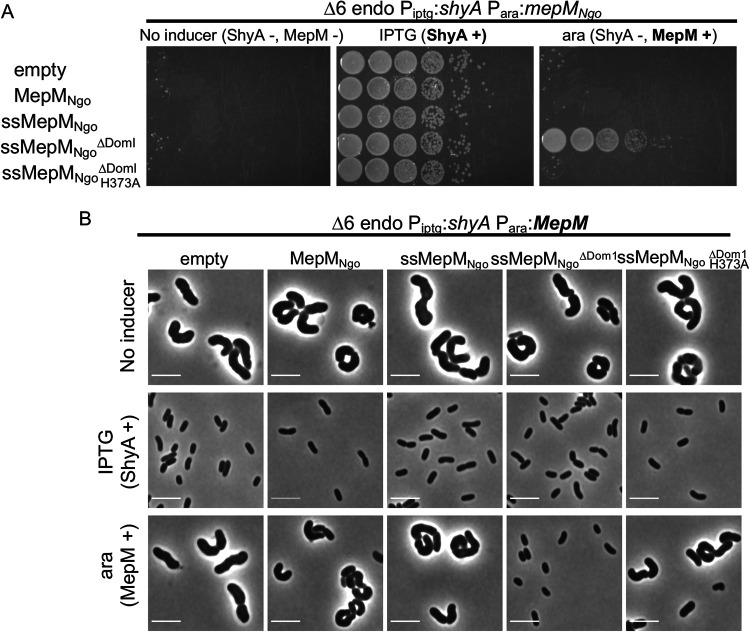
Cross-species complementation of Δ6 endo phenotypes with an EP from Neisseria gonorrhoeae. (A and B) Δ6 endo strain was transformed with (arabinose-inducible) pBAD33 expressing an N. gonorrhoeae EP (MepM_Ngo_). Derivatives of MepM_Ngo_ include an N-terminal DsbA signal sequence (ss), domain 1 truncation (ΔDom1), or active site mutation (H373A). (A) Cells were washed and spot-plated on medium containing either no inducer, IPTG (200 μM) (chromosomal ShyA +), or arabinose (0.2%) (pBAD33-encoded MepM +). Plates were incubated at 37°C for 24 h and then imaged. (B) Cells were diluted 100-fold and grown without inducer, with IPTG (ShyA +), or with arabinose (MepM +) for 3 h and then imaged. Bars, 5 μm.

So far, our results suggested that during EP insufficiency, PG synthesis via aPBPs promotes cell integrity, whereas the Rod system remains functional but is not absolutely required for mass increase or PG incorporation. It has been hypothesized that PG synthases require a physical association with one or more EPs for insertion of nascent PG material, but alternatively, EPs might catalyze PG insertion independently, for example through recognition of intrinsic PG substrate cues. To gain a better understanding of the necessity for a physical interaction, we conducted cross-species complementation experiments using an EP from Neisseria gonorrhoeae (henceforth “MepM_Ngo_”). This distantly related EP (a BLAST alignment indicated 29% identity between MepM_Ngo_ and ShyA [Fig. S4]), when heterologously expressed, is unlikely to interact with any native V. cholerae enzymes, and should thus allow us to isolate its EP activity from the interaction networks it might be embedded in. We expressed arabinose-inducible MepM_Ngo_ in Δ6 endo cells and measured differential growth in the presence of IPTG (ShyA expression) versus arabinose (MepM_Ngo_ expression). We found that wild-type MepM_Ngo_ was unable to rescue growth of Δ6 endo cells during ShyA depletion conditions (Fig. 4A). However, we recently demonstrated that EPs from diverse organisms (including E. coli and N. gonorrhoeae) are produced predominantly in an inactive form due to the inhibitory function of their domain 1 and are likely activated *in vivo* by an unknown mechanism (36). Heterologously expressed enzymes may not be subject to this activation pathway in V. cholerae (especially if the activator is a protein). We thus expressed EP mutant versions with their inhibitory domain 1 deleted (ΔDom1), rendering them constitutively active, and provided a signal sequence (ss) to ensure export to the periplasm. Surprisingly, ssMepM_Ngo_^ΔDom1^ fully complemented growth of the Δ6 endo strain to a similar degree as the native ShyA (Fig. 4A). Visual inspection of Δ6 cells relying on heterologous expression for growth (arabinose-positive [ara+] condition), revealed that complementation with ssMepM_Ngo_^ΔDom1^ (but not its active site mutant derivative H373A) promoted both growth (Fig. 4A) and the generation of rod-shaped cells (Fig. 4B). Interestingly, we found that heterologous expression of a signal sequence fusion or activated MepM from E. coli (ssMepM_Eco_ and ssMepM_Eco_^ΔDom1^, respectively) were able to restore growth, but not the rod shape, to Δ6 endo V. cholerae (Fig. S5A and B). Thus, for an unknown reason, intrinsic properties of specific EPs (or rather these mutant derivatives) define their ability to complement the Δ6 endo cell shape. We sought to confirm that this apparent complementation of the rod shape was still dependent on MepM_Ngo_^ΔDom1^ (rather than a mutation derepressing *shyA* in Δ6 endo strain). Thus, we plated all strains on agar containing IPTG, arabinose, or no inducer at the end of the experiments where we visualized cells relying on MepM_Ngo_^ΔDom1^ for growth. All strains had the same low level of spontaneous suppressors able to grow in the absence of inducer (Fig. S5C), confirming that the majority of the rod-shaped cells observed when only MepM_Ngo_^ΔDom1^ was expressed are not suppressors. In summary, these data demonstrate that heterologous expression of an activated EP can be sufficient to restore both growth and (in the case of the N. gonorrhoeae EP) proper cell shape to Δ6 endo cells.

10.1128/mBio.03596-20.8 FIG S4 ShyA and orthologous MepM amino acid sequence alignment. (A) Amino acid sequences of V. cholerae endopeptidase ShyA (VCA0079) and orthologs from N. gonorrhoeae (NGO1686) and E. coli (b1856) were aligned in Clustal Omega (74). Numbers indicate the amino acid position relative to the start codon, and alignment gaps are denoted with dashes. Symbols indicate the similarity of aligned residues: identical (*), strong similarity (:), and weak similarity (.). (B) Summary of BLAST alignments of b1656 and NGO1686 amino acid sequences to ShyA. Download FIG S4, TIF file, 0.4 MB. Copyright © 2021 Murphy et al. 2021 Murphy et al. https://creativecommons.org/licenses/by/4.0/ This content is distributed under the terms of the Creative Commons Attribution 4.0 International license.

10.1128/mBio.03596-20.9 FIG S5 Cross-species complementation of Δ6 endo strain with orthologous EPs. (A and B) Δ6 endo strain was transformed with (arabinose-inducible) pBAD33 expressing an E. coli EP (MepM_Eco_). Derivatives of MepM_Eco_ include an N-terminal DsbA signal sequence (ss), domain 1 truncation (ΔDom1). (A) Strains were diluted and spot-plated on medium containing either no inducer, IPTG (200 μM) (ShyA +), or arabinose (0.2%) (MepM +). Plates were incubated at 37°C for 24 h and then imaged. (B) Alternatively, these strains were diluted into fresh medium containing either no inducer, IPTG, or arabinose (ara) and grown for 3 h and then imaged on a 0.8% agarose pad. Scale bar, 5 μm. (C) Δ6 endo strain carrying pBAD33 expressing the indicated constructs (“pBAD” column) were diluted into fresh medium containing either no inducer, IPTG, or arabinose (ara) and grown for 3 h (“experiment” column). Cells were then spot-plated on medium containing either IPTG (200 μM) (ShyA expressed), arabinose (0.2%) (MepM expressed) or no inducer. Plates were incubated at 37 ˚C for 24 h and then imaged. Download FIG S5, TIF file, 0.6 MB. Copyright © 2021 Murphy et al. 2021 Murphy et al. https://creativecommons.org/licenses/by/4.0/ This content is distributed under the terms of the Creative Commons Attribution 4.0 International license.

## DISCUSSION

**FIG 5:**
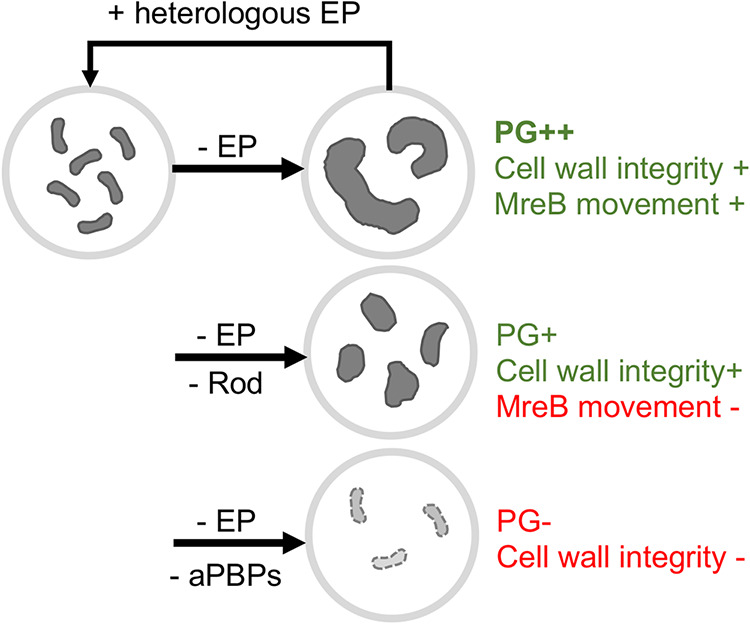
Summary diagram of the consequences of EP insufficiency in V. cholerae. Characteristics of EP-insufficient (- EP) V. cholerae in the presence or absence of the Rod system or aPBP inhibitors.

Bacteria must maintain a careful balance between cell wall cleavage and synthesis to promote cell elongation/division, but the exact relationship between the two cell wall synthases (Rod system versus aPBPs) and cell wall hydrolases (e.g., endopeptidases) is poorly understood, at least in Gram-negative bacteria. Here, we have used EP depletion and chemical inactivation experiments to dissect the interplay between cell wall cleavage and synthesis in the cholera pathogen V. cholerae. Our key observation is that in V. cholerae, cell wall synthesis and cell expansion (but not cell division) continue aberrantly upon EP depletion (Fig. 5). This poses an apparent contradiction to data obtained in E. coli, where cell wall incorporation was drastically reduced after EP depletion and cells started to lyse (23). While ostensibly fundamental aspects of the coordination between cell wall synthesis and cleavage may simply not be as well conserved as one might expect, these observations might also reflect species-specific differences in EP-independent cell wall turnover rates, and not necessarily the consequences of EP depletion *per se*. It is possible that lysis under EP-insufficient conditions in E. coli reflects generally higher PG degradation rates (E. coli, for example, encodes three amidases [59], while V. cholerae possesses only one [60]). This would mask the underlying continued incorporation of new cell wall material in the absence of EPs. Importantly, EP depletion in E. coli did result in a cell volume increase prior to lysis (23), also supporting at least a transient continuation of PG synthesis during EP insufficiency in this species. Last, it is possible that V. cholerae simply elaborates a more structurally robust cell envelope than E. coli, which seems ecologically appropriate given the wide range of osmolarities (from pond water to seawater) the cholera pathogen resides in.

The amount of cell wall expansion occurring during EP insufficiency was surprising, since presumably any form of cell wall synthesis that promotes the degree of cell expansion we observed in EP-deficient V. cholerae might be expected to require some form of cleavage. This cleavage is likely catalyzed by other autolysins; however, the incisions resulting from such cleavage, and/or the autolysin(s) involved, appear to be of limited utility to the Rod system, while they can be exploited by the aPBPs. This suggests that aPBPs are more versatile in recognizing a variety of cell wall cuts required for structural integrity (independent of an actual physical connection with EPs), while the Rod system primarily relies on either EP-mediated cleavage or a physical association with EPs (see a more detailed discussion below) to promote proper cell elongation and width homeostasis. These interpretations are in line with some old and several recent proposals based on data from E. coli that aPBPs and SEDS have separate (yet perhaps overlapping) functions during cell elongation (15, 61, 62). Interestingly, upregulated EP activity promotes aPBP function in E. coli, likely indirectly through the creation of PG incisions that allow for an interaction between aPBPs and their OM-localized activators (30). Thus, EP cleavage may not be strictly necessary for, but can promote, aPBP activity. It is possible that under EP-insufficient conditions, the lytic transglycosylases (the other major group of cell wall cleavage enzymes that cut the polysaccharide backbone of PG [63]) create large open areas in PG that can be recognized, and patched, by aPBPs; however, undiscovered EPs might also play a role.

The observation (consistent with what has been shown in B. subtilis [24]) that MreB continues directed movement at least for some time during EP insufficiency suggests that the Rod system does not actually require wild-type EP activity for RodA’s glycosyltransferase activity (which likely drives MreB movement). This suggests that EPs may not be a functionally integral part of the Rod system in V. cholerae. Similar to what has been proposed for B. subtilis, it is thus tempting to speculate that V. cholerae may use a “make-before-break” model as proposed by Höltje (38) and Koch (37) for cell elongation via the Rod system. In this model, the Rod system creates a second layer of PG that is incorporated via EPs during or after synthesis. Generation of this second layer could at first proceed independently of wild-type EP activity, but incorporation into the growing sacculus would require cross-link cleavage.

The results of our cross-species complementation experiments with an activated N. gonorrhoeae EP further suggest that a physical association between the Rod system and EPs might not be strictly necessary, unless the heterologously expressed (and truncated) enzyme does somehow directly interact with the V. cholerae Rod system. We thus consider a model plausible where rather than (or in addition to) coordinating with cell wall synthases directly, EPs can somehow specifically recognize and preferentially cleave old PG that is adjacent to nascent PG. Though highly speculative, our observation that the corresponding E. coli homolog does not complement cell shape might reflect different levels of activity—since EPs can promote aPBP activation (at least in E. coli) (30), overexpression of a more active EP might divert PG precursor flux away from the Rod system toward aPBPs to a higher degree than the N. gonorrhoeae enzyme, incapacitating the cells’ ability to elaborate a rod shape.

An important caveat to the complementation experiments is that the Δ6 endo strain still maintains a copy of *shyA* under IPTG control. While the *lac* promoter is tightly repressed in the absence of inducer, a small number of molecules under its control might still be produced (64). ShyA is produced predominantly as an inactive precursor, and the signal for activation is unknown (36). It is conceivable that complementation with a heterologously expressed EP might somehow enhance activation of this leaky background of ShyA molecules, if there is, for example, a positive feedback loop between cell wall cleavage and native EP activation.

In summary, our data suggest that two main cell wall synthases, the aPBPs and the Rod system have differential relationships with autolysins, and especially endopeptidases. As such, our data provide additional support for the emerging theme of at least partially differential roles of the aPBPs and the Rod system during cell elongation.

## MATERIALS AND METHODS

### Bacterial growth conditions.

Cells were grown with shaking (200 rpm) at 37°C in 5 ml of LB in borosilicate glass tubes (14-ml capacity) unless otherwise indicated. Where appropriate, antibiotics were used at the following concentrations: streptomycin, 200 μg ml^−1^; ampicillin, 100 μg ml^− 1^; chloramphenicol, 20 μg ml^−1^; moenomycin, 10 μg ml^−1^; MP265, 300 μM; amdinocillin, 10 μg ml^−1^; and cefsulodin, 1 mg ml^−1^. IPTG (200 μM) and arabinose (0.2%) were added for induction of P_IPTG_ and P*_ara_* promoters, respectively.

### Plasmid and strain construction.

All bacterial strains and oligonucleotides used in this study are summarized in Table S1 in the supplemental material. All Vibrio cholerae strains are derivatives of El Tor strain N16961 (65) or E7946 (66); the latter strain was used for chitin-induced transformation.

10.1128/mBio.03596-20.1 TABLE S1 Summary of strains and oligonucleotides used in this study. Download Table S1, XLSX file, 0.01 MB. Copyright © 2021 Murphy et al. 2021 Murphy et al. https://creativecommons.org/licenses/by/4.0/ This content is distributed under the terms of the Creative Commons Attribution 4.0 International license.

Construction of Δ6 endo strain is reported elsewhere (32). Other strains were constructed by chitin-induced transformation of linear PCR products as described in reference 67. A chloramphenicol (*chl*) resistance cassette insertion into the gene *vc1807* (a well-established neutral locus) was used as the primary selector. The transforming fragment for *vc1807*::*chl* was constructed by amplifying upstream and downstream homology regions using primers PD079/PD097 and PD098/PD082, respectively. The *chl* gene coding for chloramphenicol acetyltransferase was amplified from pBAD33 (68) with primers PD095/PD096 and fused with the flanking homologies of *vc1807* via isothermal assembly. For antibiotic resistance gene swapping, a *vc1807*::trim allele was also produced by amplifying upstream (using primers TDP597/598) and downstream (primers TDP601/602) homologies of *vc1807* and fusing them with a trimR cassette amplified from V. cholerae Haiti (69) (primers TDP599/600) using splicing by overlap extension PCR (SOE PCR) with primers TDP603/604.

To construct a functional MreB-msfGFP-MreB sandwich fusion, upstream (primers PD056/PD074) and downstream (primers PD071/PD057) homologies were amplified from the V. cholerae genome and fused via isothermal assembly with monomeric superfolder green fluorescent protein (msfGFP) (amplified with primers PD054/PD055). Analogous to a published E. coli MreB-msfGFP sandwich fusion (52), we replaced glycine 228 of MreB with this msfGFP. To enhance the probability of success of finding a functional fusion, we used semidegenerate primers to generate a library of possible linker sequences. Flanking homologies, MreB and msfGFP were first fused using isothermal assembly (70) and then amplified using nesting primers PD104/PD105. The resulting upstream-MreB-linker-msfGFP-linker-MreB-downstream PCR fragments were transformed into E7946 cells using chitin transformation with *vc1807*::*chl* as the primary selector. Ninety-six colonies were tested for growth rate, and clone M2C was chosen for further experiments due to its wild-type growth behavior. The linkers of this fusion construct were sequenced (coding for DGVGG upstream of msfGFP and GTPIP downstream).

Δ8 strain was constructed by transforming endopeptidase deletion PCR products into a parental *mreB*::*mreB*msfGFP Δ*lacZ*::P_IPTG_::*shyA* strain. Deletion scars were amplified from Δ6 endo strain and introduced in two steps into this parental background via chitin transformation. The following primers were used to amplify the EP deletion fragments: *shyA* (TDP577/578), *shyC* (TDP581/582), *shyB* (TDP579/580), *vc1537* (TDP583/584), *vc0843* (tagE1) (TDP587/588), and *vca1043* (tagE2) (TDP585/586). PBP4 and PBP7 deletions were introduced into Δ6 endo strain by amplifying PCR fragments with upstream and downstream homologies fused by a linker for chitin-mediated transformation. PBP4 upstream homology (TDP680/TDP681) and downstream homology (TDP682/TDP683) were fused using SOE PCR with nesting primers (TDP691/TDP692). PBP7 upstream homology (TDP676/TDP677) and downstream homology (TDP678/TDP679) were fused using SOE PCR with nesting primers (TDP693/TDP694). For the Δ*ldtA* Δ*ldtB* strain, deletion scars were amplified from strain FC670 (42) using primers TDP654/55 (*vc1268*) and TDP656/57 (*vca0058*), respectively, and transformed into Δ6 endo strain using chitin transformation as described above.

Δ8 strain exhibited a very low transformation efficiency, and we thus introduced the Δ*vc1269* deletion using homologous recombination with a suicide plasmid, pCVD442, as described previously (71). In brief, upstream and downstream homologies of vc1269 were amplified using primers TD810/TD811 and TD812/TD813. These fragments were cloned into XbaI-digested pCVD442 using isothermal assembly. pCVD442(Δvc1269) was then introduced into the Δ8 endo strain via biparental mating (using SM10 as a donor strain) by mixing 10 μl of each donor and recipient, followed by 6 h of incubation at 37°C, followed by selection for single crossover strains and against the donor strain by plating on LB plates containing carbenicillin (100 μg ml^−1^), streptomycin (200 μg ml^−1^), and IPTG (200 μM). A single colony from the first crossover plate was then picked and streaked out on a plate containing sucrose (10%), streptomycin (200 μg ml^−1^), and IPTG (200 μM). This plate was incubated at ambient temperature for 3 days, after which 16 colonies were tested for the correct knockout construct using *vc1269*-flanking primers TD814/TD815.

All plasmids were built using isothermal assembly (70). Genes were cloned into pBADmob (a mobile pBAD33 derivative) using the following primer pairs: MepM_Ngo_, TDP1365/TDP1367, ssMepM_Ngo_, SM861/SM862; ssMepMNgo^Δdom1^, SM859/SM860; MepM from E. coli (MepM_ECO_), TDP1342/TDP1340; MepM_ECO_, TDP1339-B/TDP1340; and MepM_ECO_^Δdom1^, TDP1341/TDP1340. The H373A point mutation was introduced into pBAD plasmids carrying NGO1686 derivatives via Q5 site-directed mutagenesis (New England BioLabs [NEB], Ipswitch, MA; catalog no. E0554S) with primer pair TDP1652/TDP1653. Plasmids were conjugated into V. cholerae using donor E. coli strains (SM10 lambda pir or MFD lambda pir).

### Endopeptidase depletion experiments.

EP depletion strains were grown overnight in LB broth containing 200 μM IPTG. The next day, cells were washed two times by pelleting (2 min at 13,400 × *g*) and resuspended in LB broth without inducer. Cells were then diluted 100-fold into fresh LB containing either 200 μM IPTG (with ShyA [ShyA +]) or no inducer (without ShyA [ShyA -]). After 2 hours of depletion, antibiotics were used at 10 μg ml^−1^ (moenomycin, amdinocillin), 300 μM (MP265), or 1 mg ml^−1^ (cefsulodin) where indicated.

### Bacterial dry weight.

One hundred twenty milliliters of culture was harvested (4,830 × *g*, 10 min), resuspended in ∼1 ml, and transferred to a preweighed 2-ml centrifuge tube. Samples were pelleted at maximum speed (16,100 × *g*, 2 min) again to remove any residual supernatant. Tubes were incubated on a heat block at 65°C with the lids open until the pellets were completely dry and mass measurements stabilized (∼24 h). Values were transformed to milligram per liter (mg/liter) units.

### Phase contrast microscopy and HADA staining.

Cells were harvested (2 min at 13,400 × *g*), spotted on a 0.8% agarose pad containing phosphate-buffered saline (PBS) and imaged on a Leica Dmi8 inverted microscope. Cell dimensions (area, length, and width) were quantified using the particle analysis function in ImageJ (50). For HADA experiments, Δ6 endo cells were grown in the presence of 50 μM HADA (3-[[(7-hydroxy-2-oxo-2*H*-1-benzopyran-3-yl)carbonyl]amino]-d-alanine hydrochloride), washed once by pelleting cells (2 min at 13,400 × *g*) and resuspended in fresh LB. HADA stain was imaged in the 4′,6′-diamidino-2-phenylindole (DAPI) channel (395 nm [excitation]/460 nm [emission]) at 1-s exposure.

### Single particle tracking by TIRF imaging.

The Δ8 endo *mreB*::*mreB*msfGFP^sw^ strain with chromosomally expressed MreB-msfGFP was grown shaking at 37°C in LB medium supplemented with 100 μM IPTG overnight. The saturated cells were diluted (1:100) into fresh LB in two groups (with 100 μM IPTG for ShyA expression or without IPTG for ShyA depletion). After 2 h of shaking (220 rpm) incubation at 37°C, cells were harvested and spotted on a 0.8% agarose pad containing M9 medium. Time-lapse TIRF (total internal reflection fluorescence) imaging was performed on a Zeiss Elyra equipped with an inverted Axio Observer.Z1 microscope and a 100×  oil objective (numerical aperture, 1.46). The objective was heated at 37°C during image acquisition. The exposure time was 100 ms, and interframe intervals were 2 s over a 2-min recording. The measurement of cluster size and number was performed using ImageJ TrackMate (54, 57, 58). The background of fluorescent images was subtracted using *Image Calculator* to reduce background noise. A threshold range was set to distinguish the objects of interest from the background and convert the image to binary (via *Adjust Threshold*). Automatic particle analysis was performed using *Analyze Particles*. The mean square displacements (MSD) of particle trajectories were calculated using the msdanalyzer package, and the motion types were analyzed through log-log fitting (55). By setting the *R*^2^ coefficient > 0.8, individual MSD curves were fitted, and the values of anomalous diffusion coefficient (α) indicate that MreB particles exhibit a mix of dynamic behaviors (confined diffusion, 0.1 ≤ α < 0.9; simple diffusion, 0.9 ≤ α < 1.1; directed motion, α ≥ 1.1) (72).

### Peptidoglycan analysis.

PG samples were analyzed as described previously (73). Briefly, 50-ml cultures of Δ6 endo strain were grown to early/mid exponential phase with or without IPTG (200 μM) for 2 h, harvested, and boiled in 5% sodium dodecyl sulfate (SDS) for 1 h. Sacculi were repeatedly washed by ultracentrifugation (150,000 × *g*, 10 min, 20°C) with MilliQ water until SDS was totally removed. Samples were treated with 20 μg proteinase K (1 h, 37°C) for Braun’s lipoprotein removal and finally treated with muramidase (100 μg ml^−1^) for 16 h at 37°C. Muramidase digestion was stopped by boiling, and coagulated proteins were removed by centrifugation (22,000 × *g*, 10 min). For sample reduction, the pH of the supernatants was adjusted to pH 8.5 to 9.0 with sodium borate buffer, and sodium borohydride was added to a final concentration of 10 mg ml^−1^. After incubating for 30 min at room temperature, the pH of the samples was adjusted to pH 3.5 with orthophosphoric acid.

Ultrahigh-performance liquid chromatography (UPLC) analyses of muropeptides were performed on a Waters UPLC system (Waters Corporation, USA) equipped with an Acquity UPLC ethylene bridged hybrid (BEH) C_18_ column (130 Å, 1.7 μm, 2.1 mm × 150 mm) (Waters, USA) and a dual wavelength absorbance detector. Elution of muropeptides was detected at 204 nm. Muropeptides were separated at 45°C using a linear gradient from buffer A (0.1% formic acid in water) to buffer B (0.1% formic acid in acetonitrile) in an 18-min run, with a 0.25-ml/min flow.

Relative total PG amount was calculated by comparison of the total intensities of the chromatograms (total area) from three biological replicas normalized to the same OD_600_ and extracted with the same volumes. Muropeptide identity was confirmed by tandem mass spectrometry (MS/MS) analysis, using a Xevo G2-XS quadrupole time of flight (QTof) system (Waters Corporation, USA). Quantification of muropeptides was based on their relative abundances (relative area of the corresponding peak) normalized to their molar ratio.

### Western blotting.

Whole-cell lysates (15 μg) were resolved by 10% SDS-polyacrylamide gel electrophoresis (PAGE), and the proteins were transferred to a polyvinylidene difluoride (PVDF) membrane using a semidry transfer system (iBlot 2; Invitrogen). The membrane was then blocked overnight with blocking solution containing 4% milk (dry milk dissolved in 20 mM Tris-HCl [pH 7.8], 150 mM NaCl, 0.1% Triton X-100). The next day, the membrane was incubated with anti-ShyA polyclonal antibody (1:5,000) (produced by Pocono Rabbit Farm & Laboratory, PA) for 2 h and then washed twice with 1× TBST (20 mM Tris-HCl [pH 7.8], 150 mM NaCl, 0.1% Triton X-100). The washed membranes were then incubated with anti-rabbit secondary antibody (1:15,000) (Li-Cor catalog no. 926-32211) for 1 h. Membranes were then washed three times with 1× TBST, scanned on an Odyssey CLx imaging device (Li-Cor Biosciences) and visualized using Image Studio Lite version 5.2 software (Li-Cor) for signal quantification.

10.1128/mBio.03596-20.4 MOVIE S3 mreBmsfGFP movement in Δ6 endopeptidase expressing *shyA*. Time-lapse movie showing movement of MreBmsfGFP^sw^ particles. Images were taken every 5 s at 100-ms exposure time. The movie is minimally processed (ImageJ’s walking average function). Download Movie S3, AVI file, 0.1 MB. Copyright © 2021 Murphy et al. 2021 Murphy et al. https://creativecommons.org/licenses/by/4.0/ This content is distributed under the terms of the Creative Commons Attribution 4.0 International license.
