# Supplementary figures and images for: Class A Penicillin-Binding Protein-Mediated Cell Wall Synthesis Promotes Structural Integrity during Peptidoglycan Endopeptidase Insufficiency in Vibrio cholerae
Source: mBio. 2021 Apr 6;12(2):e03596-20. doi: 10.1128/mBio.03596-20 (PMC8092314; doi:10.1128/mBio.03596-20)

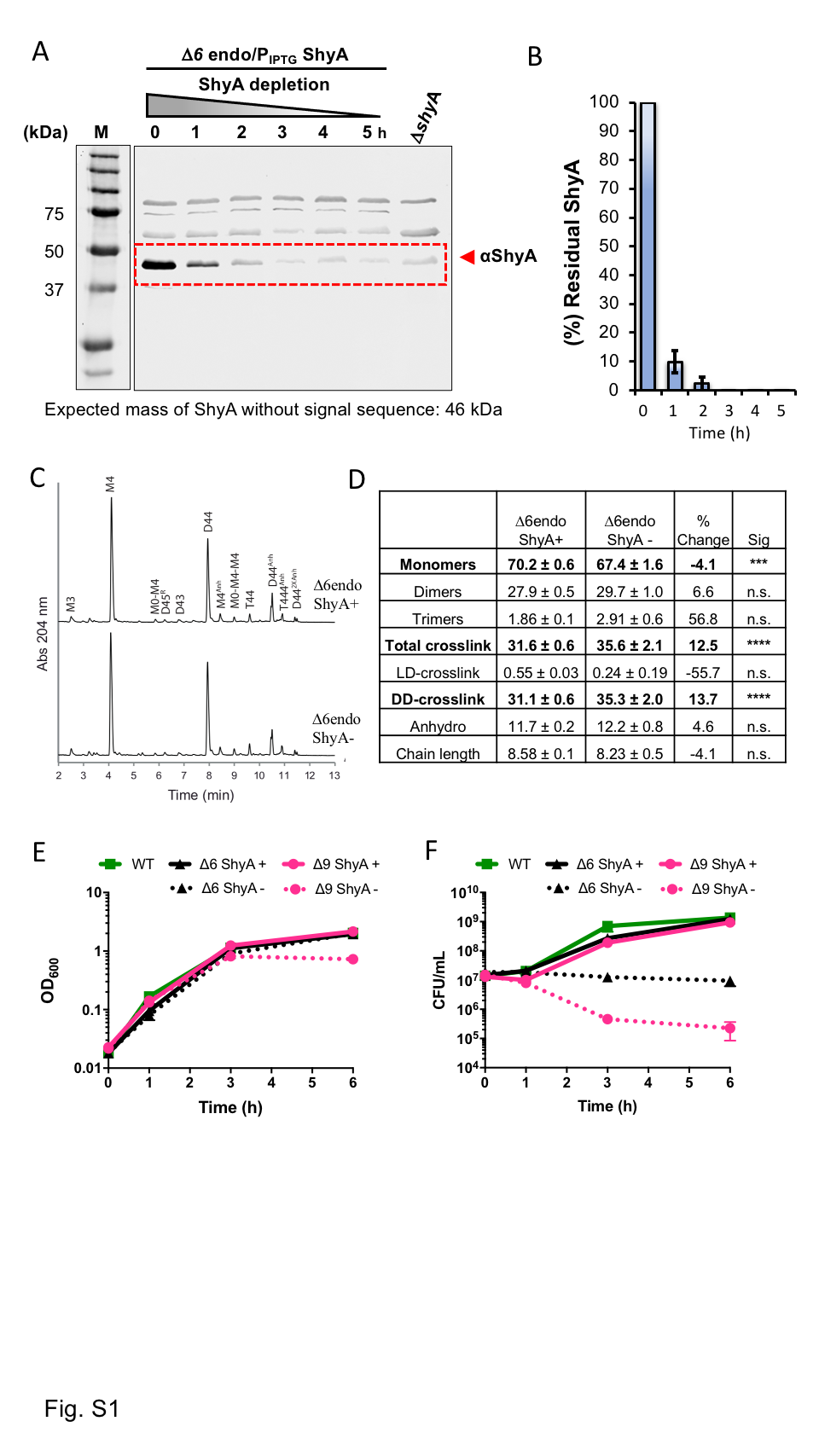

Supplement: FIG S1 [file mBio.03596-20-sf001.tif]

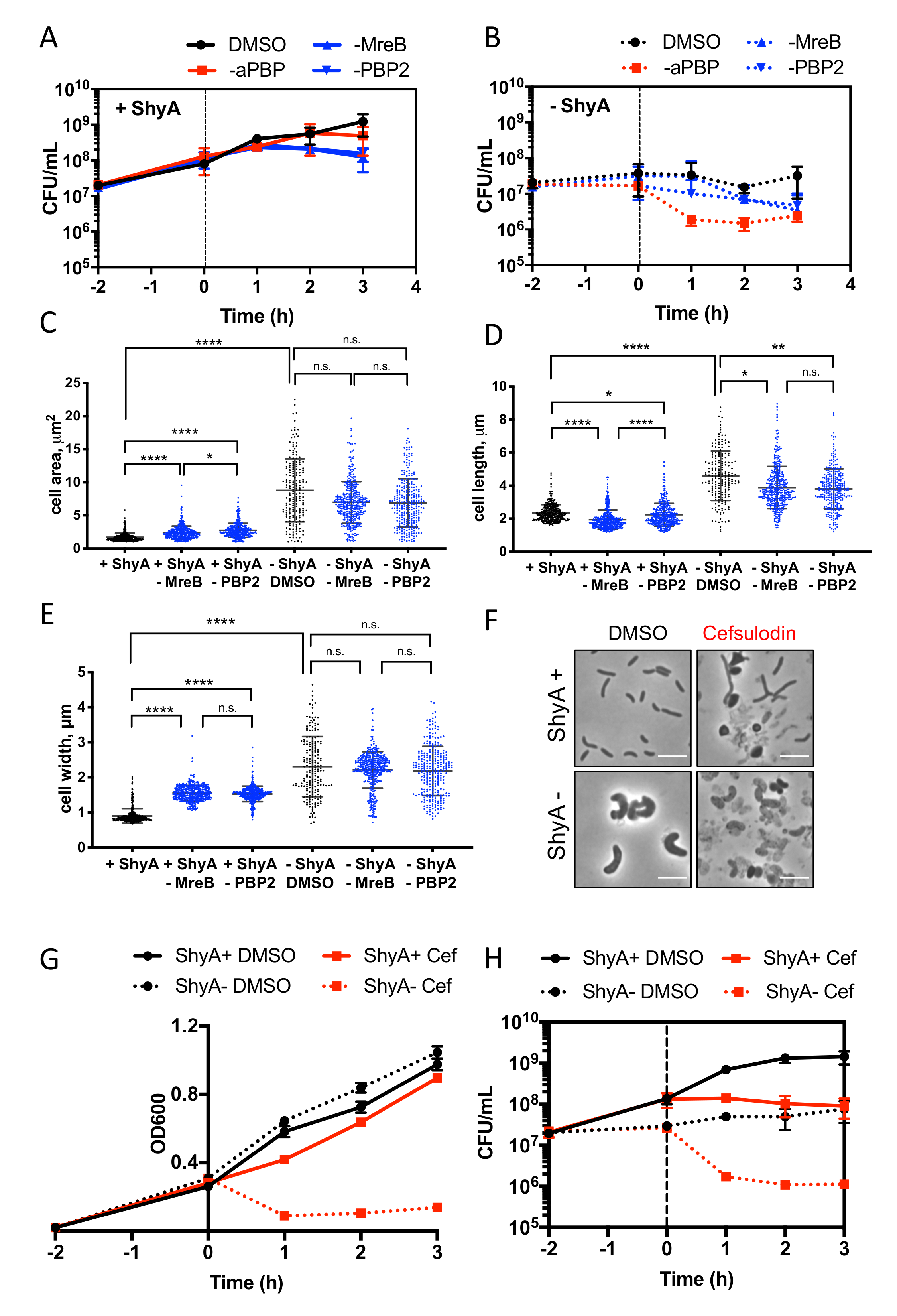

Supplement: FIG S2 [file mBio.03596-20-sf002.tif]

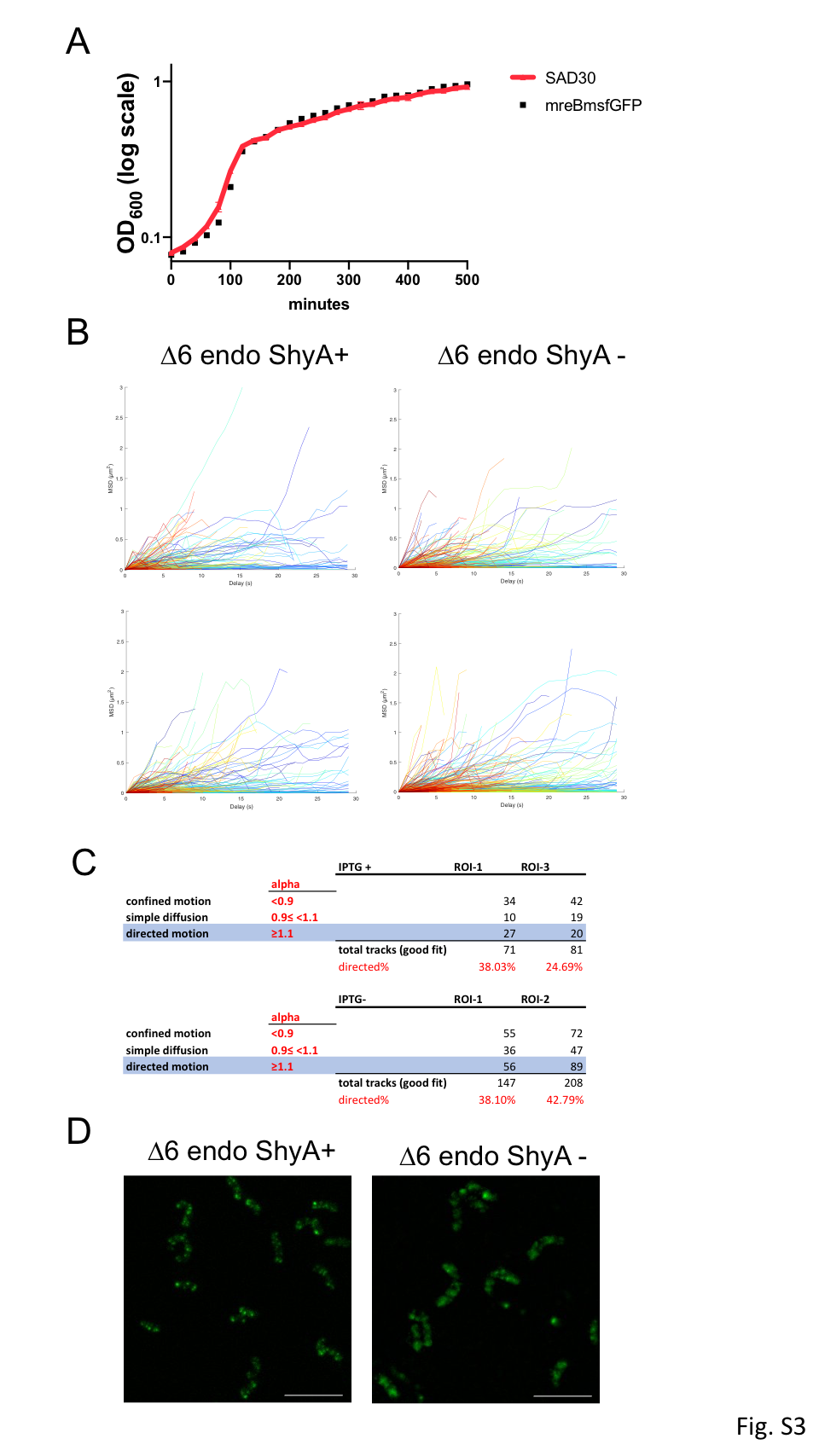

Supplement: FIG S3 [file mBio.03596-20-sf003.tif]

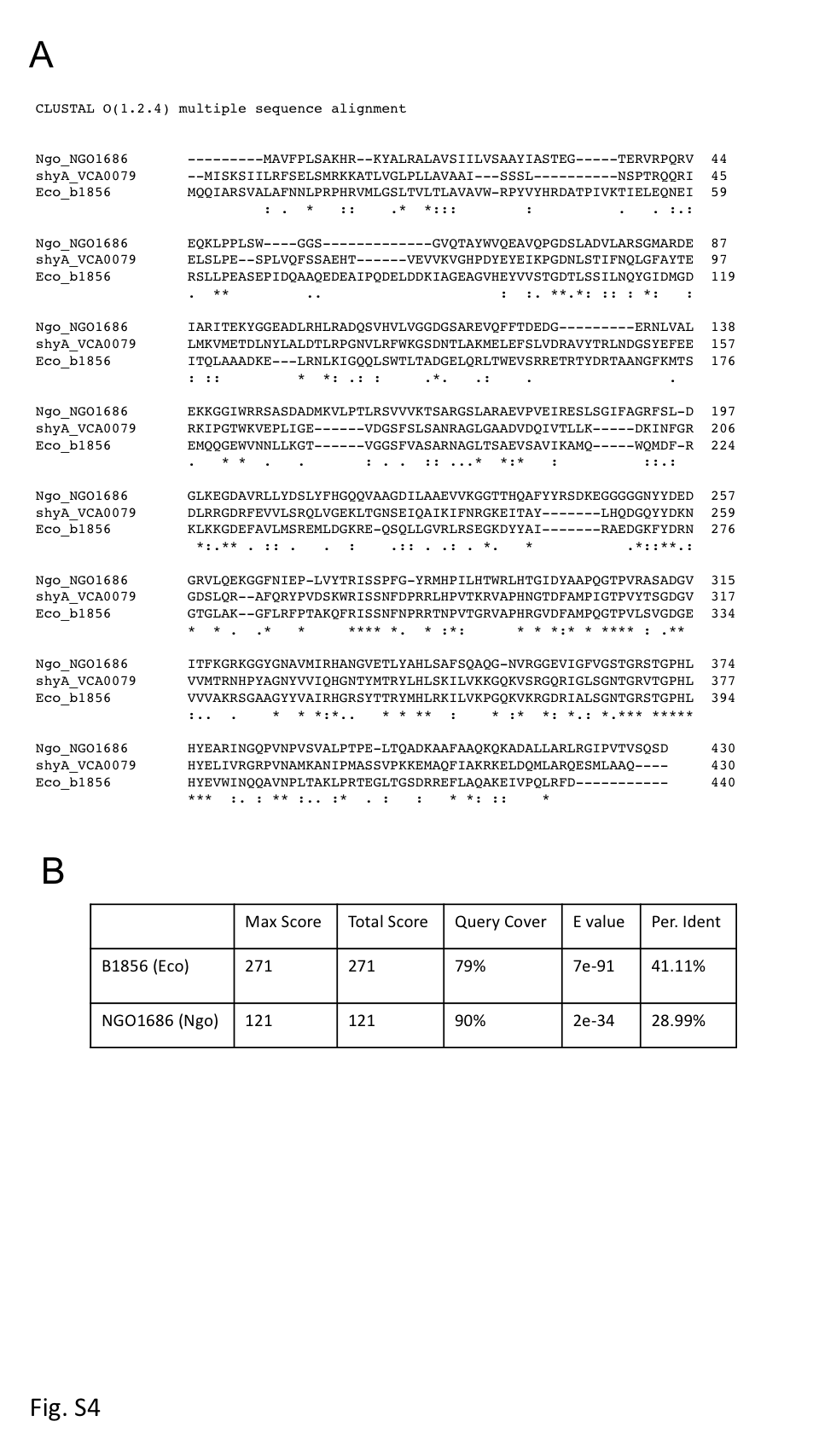

Supplement: FIG S4 [file mBio.03596-20-sf004.tif]

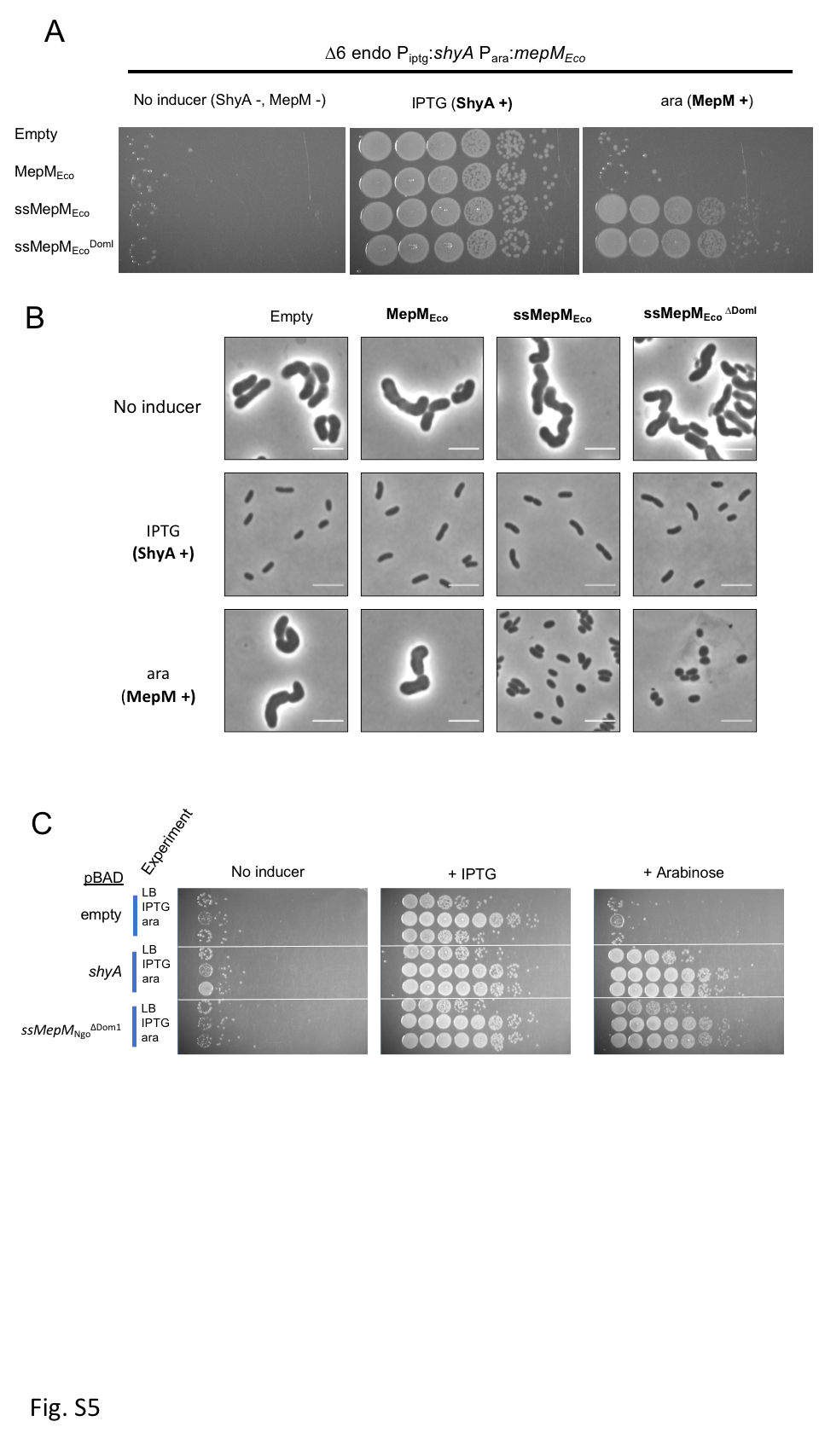

Supplement: FIG S5 [file mBio.03596-20-sf005.tif]
